# Use of Poly (Amidoamine) Dendrimer for Dentinal Tubule Occlusion: A Preliminary Study

**DOI:** 10.1371/journal.pone.0124735

**Published:** 2015-04-17

**Authors:** Tianda Wang, Sheng Yang, Lei Wang, Hailan Feng

**Affiliations:** 1 Department of Prosthodontics, Peking University School and Hospital of Stomatology, Beijing, China; 2 Chongqing Key Laboratory of Oral Diseases and Biomedical Sciences, College of Stomatology, Chongqing Medical University, Chongqing, China; Universidad de Castilla-La Mancha, SPAIN

## Abstract

The occlusion of dentinal tubules is an effective method to alleviate the symptoms caused by dentin hypersensitivity, a significant health problem in dentistry and daily life. The *in situ* mineralization within dentinal tubules is a promising treatment for dentin hypersensitivity as it induces the formation of mineral on the sensitive regions and occludes the dentinal tubules. This study was carried out to evaluate the *in vitro* effect of a whole generation poly(amidoamine) (PAMAM) dendrimer (G3.0) on dentinal tubule occlusion by inducing mineralization within dentinal tubules. Dentin discs were treated with PAMAM dendrimers using two methods, followed by the *in vitro* characterization using Attenuated total reflection Fourier-transform infrared spectroscopy (ATR-FTIR), X-ray diffraction (XRD), Field emission scanning electron microscopy (FE-SEM) and Energy-Dispersive X-ray Spectroscopy (EDS). These results showed that G3.0 PAMAM dendrimers coated on dentin surface and infiltrated in dentinal tubules could induce hydroxyapatite formation and resulted in effective dentinal tubule occlusion. Moreover, crosslinked PAMAM dendrimers could induce the remineralization of demineralized dentin and thus had the potential in dentinal tubule occlusion. In this *in vitro* study, dentinal tubules occlusion could be achieved by using PAMAM dendrimers. This could lead to the development of a new therapeutic technique for the treatment of dentin hypersensitivity.

## Introduction

Dentin hypersensitivity is one of the most commonly occurring clinical dental conditions which is characterized by short, sharp pains and arises from exposed dentin reacting to external stimuli, which typically are thermal, evaporative, tactile, osmotic or chemical[1,2]. Hydrodynamic theory, the most generally accepted theory regarding the mechanism of dentin hypersensitivity proposes that a pain-provoking stimulus increases the flow of the dentinal tubular fluid, or changes the flow direction, and consequently, stimulates the nerves around the odontoblasts, leading to dentin hypersensitivity[3]. Based on the hydrodynamic theory, two main strategies: (1) occluding the dentinal tubules to reduce the fluid flow, and (2) decreasing the excitability of the intradental nerve by the application of chemical agents containing potassium ions are therefore the common approaches for treating dentin hypersentitivity[4,5]. Dentin desensitizing products, containing agents such as fluoride, strontium salts, oxalate, glutaraldehyde and bioactive glass, are employed to treat dentin hypersensitivity[6–10]. Although these products and agents were reported to be effective, short durability and poor effectiveness were often exhibited as the therapeutic effects of these desensitizing products were short lived on account of daily tooth brushing or drinking of acidic beverages and the occlusion effects were sometimes incomplete[11,12]. Therefore, new methods that can solve these problems need to be figured out. Hydroxyapatite (HAP) is thus an ideal candidate.

As the main mineral component of human hard tissues such as bone, enamel and dentin, HAP is an ideal material evoking considerable interest in the occlusion of dentinal tubules, on account of the abilities of HAP in inducing natural response of cells and further mineralization of precipitate with calcium and phosphate existing in saliva or pulpal fluid[13]. Although acceptable occlusion effects could be achieved by employing nano-hydroxyapatite (nano-HAP) as desensitizing agent, the particle size of nano-HAP and the weak intercrystalline binding affinity would limit the direct application of HAP on occluding dentinal tubules. When used as an additive of dentifrice, the effects of HAP might also be minimized because of the interference resulted from other ingredients in the dentifrice[14]. The *in situ* formation of HAP for the occlusion of dentinal tubules in a variety of ways for example the application of calcium phosphate precipitate method (CPP method) or mesoporous biomaterials was thus discussed[11,13,15–18]. Due to the experimental results obtained by these researches, the application of *in situ* formed HAP is a promising option for the treatment of dentin hypersensitivity.

Dendritic structures which can be found at all dimensional length scales all over the nature world and possess unique interfacial and functional performance advantages are now being imitated by incessantly growing technologies. Among the numerous achievements, the successful synthesis of dendrimers is undoubtedly the most conspicuous one. Poly(amidoamine) (PAMAM) dendrimers, synthesized by Tomalia et al. with divergent method, were the first commercialized and extensively investigated complete dendrimer family[19]. Due to their unique and well-defined secondary structures, PAMAM dendrimers have been proposed as mimics of proteins and considered to be good candidates for studying inorganic crystallization, particularly the formation of calcium phosphate and hydroxyapatite as the morphology and size of hydroxyapatite were demonstrated to be regulated under hydrothermal conditions assisted with amine or carboxylic acid terminated PAMAM dendrimers[20,21]. Besides the functional groups which could affect the process of crystallization, the layered structures of PAMAM dendrimers also make them easily modifiable in order to tailor the properties to match various requirements[19,22].

The aim of this study was to evaluate the ability of PAMAM dendrimer (G3.0) in inducing dentinal tubule occlusion. In the present study, two different experimental approaches were carried out to determine whether the formation of HAP could be induced in dentinal tubules in the presence of PAMAM dendrimer. The null hypothesis tested was that whole generation PAMAM dendrimer could not initiate the *in situ* nucleation of HAP crystals and then totally occlude dentinal tubules.

## Materials and Methods

### Preparation of dentin discs

Extracted caries-free human third molars were collected, after obtaining written informed consent, under a protocol approved by the Ethics Committee of Peking University School and Hospital of Stomatology. The teeth were cleaned thoroughly and stored in 0.5% thymol at 4°C for no longer than one month prior to their use[23].

Dentin discs, each with a thickness of approximately 1.0 mm, were prepared by making two parallel cuts perpendicular to the long axis of each tooth, above the cement-enamel junction (CEJ), using a low-speed water cooled diamond saw (Isomet, Buehler Ltd., Lake Bluff, IL, USA). Each disc was carefully prepared and inspected to ensure that they were free of coronal enamel or pulpal exposures.

### Experimental design

After the preparation of the specimens, the coronal surfaces of the dentin discs were polished with 400, 800 and 1,200 grit carbide polishing papers under running water. The smear layer was removed by means of ultrasonication using an ultrasonic cleaner (FS20, Fisher Scientific Co., Pittsburgh, PA, USA) in distilled water for 30 s. The dentin discs were equally distributed into groups each containing thirty-two specimens and subgroups each containing sixteen specimens. In order to determine the capacities of PAMAM dendrimer on the formation of HAP in dentinal tubules, two different experimental approaches were carried out.

- Group 1 (Control 1): specimens were treated with 10% citric acid for 30 seconds and rinsed with deionised water.
- Group 2: citric acid treated specimens (same with Group 1) were coated with pure G3.0 PAMAM dendrimer (CY Dendrimer Technology Ltd., Weihai, China) for 30 seconds and rinsed with deionised water.
- Group 3 (Control 2): specimens were demineralized with 0.5 M neutral EDTA solution at room temperature for 72 hours under shaking and rinsed with deionised water.
- Group 4: EDTA demineralized specimens (same with Group 3) were treated with PAMAM-glutaraldehyde solution (0.25% glutaraldehyde aqueous solution which is containing 0.5% G3.0 PAMAM dendrimer) at 4°C for 24 hours and rinsed with deionised water.

Simulated body fluid (SBF) containing 2.5 mmol/L Ca^2+^ and 1.0 mmol/L HPO_4_^2-^ (Table 1) was used in the subsequent experiments because the main components and ionic strength of SBF were very similar to those of human saliva. The pH of SBF was adjusted to pH 7.25 at 36.5°C, by using 50 mmol/L of tris(hydroxymethyl)aminomethane and approximately 45 mmol/L of HCl. Induced mineralization experiments were performed by immersing each specimen into 50 mL of SBF at 37°C for 7 days under shaking. SBF was changed every two days. To characterize the effects of PAMAM dendrimer on the formation of HAP and the occlusion of dentinal tubules, these dentin discs were first checked by Attenuated total reflection Fourier-transform infrared spectroscopy (ATR-FTIR) and X-ray diffraction (XRD) and were then characterised by Field emission scanning electron microscopy (FE-SEM) and Energy-Dispersive X-ray Spectroscopy (EDS).

**Table 1:** Composition of Simulated Body Fluid (SBF).

Composition	Concentration (mmol/L)
NaCl	136.8
NaHCO_3_	4.2
KCl	3.0
K_2_HPO_4_	1.0
MgCl_2_	1.5
CaCl_2_	2.5
Na_2_SO_4_	0.5
Na_3_N	3.08

### Characterisation

#### ATR-FTIR analysis

An infrared spectrophotometer (NICOLET 6700, Thermo Scientific Inc. Waltham, MA, USA) with a diamond attenuated total reflection (ATR) set-up was used to collect FTIR spectra from dentin discs with different treatments. In addition, to determine the PO_4_^3-^ adsorption properties of treated and untreated dentin discs, three discs from each group was immersed in phosphate buffer solution (PBS, pH = 5.8) containing 0.184 mol/L NaH_2_PO_4_ and 0.016 mol/L Na_2_HPO_4_ for 2 hours. In the acidic conditions, amino groups of PAMAM dendrimer would be allowed to obtain a much stronger electron affinity due to the protonation of them. Spectra were collected in the range from 400 to 4,000 cm^-1^ at a 4 cm^-1^ resolution, with 32 scans.

#### XRD measurement

XRD measurements were carried out with an X-ray diffractometer (D/MAX 2400, Rigaku, Japan) to detect the crystal orientation and mineral phase of the newly formed crystals. The data were collected in the 2θ range from 15–50 degrees at a scan rate of 2 degrees per minute.

#### SEM observation and EDS analysis

After immersed in SBF for 7 days, the dentin discs were rinsed three times with deionized water, dehydrated using an ascending ethanol series (70–100%), and then, as the final chemical dehydration step, immersed in hexamethyldisilazane, which was allowed to evaporate slowly. The surface topography of the dehydrated dentin discs was observed by SEM (JSM-6301F, JEOL, Japan) with a beam voltage of 15 kV. The vertical sections were also observed after the dentin discs were split perpendicularly to the treated surfaces. An energy-dispersive X-ray spectroscopy (EDS) apparatus attached was used to identify elemental analysis on the dentin surface treated with different methods.

## Results

### Fourier-Transform infrared spectroscopic analysis

**Fig 1:**
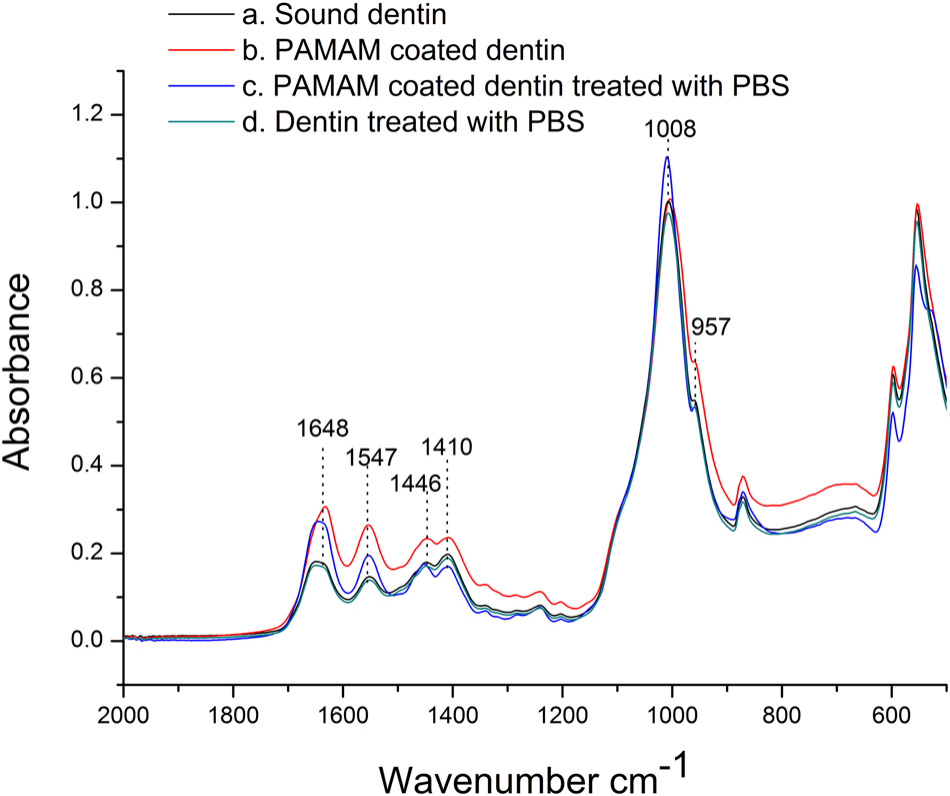
Infrared spectra (2,000–500 cm^-1^) of PAMAM coated dentin discs. (a) Sound dentin (black line). (b) Dentin disc coated with pure G3.0 PAMAM dendrimers for 30s (red line). (c) Dentin disc immersed in pH 5.8 PBS for 2h after it was coated with G3.0 PAMAM dendrimers (blue line). (d) Untreated dentin disc immersed in pH5.8 PBS for 2h (green line).

ATR-FTIR spectra of PAMAM dendrimer coated samples are shown in Fig 1. After dentin discs were coated with pure G3.0 PAMAM dendrimer, the resonances at 1648 and 1547 cm^-1^, assigned as amide I and amide II bands became more intense (b), compared with sound dentin (a), indicating that dendrimer molecules were successfully attached to the surface of dentin discs. After PAMAM coated dentin discs were treated with PBS, the resonances around 1000 cm^-1^ (1008 and 957 cm^-1^), attributing to PO_4_^3-^ were strengthened and the resonances assigned as amide I and amide II bands were weakened (c), while such performances didn’t appeared after dentin discs were immersed in PBS directly without treated with PAMAM dendrimer (d). These results indicated that the PAMAM dendrimer molecules attached to the surface of dentin discs had the capacity to attract phosphate radicals (PO_4_^3-^) through electrostatic interaction. Resonances corresponding to CO_3_^2-^ vibration at 1410 and 1446 cm^-1^ revealed the existence of carbonate-substituted hydroxyapatite as the main component of dentin[24].

**Fig 2:**
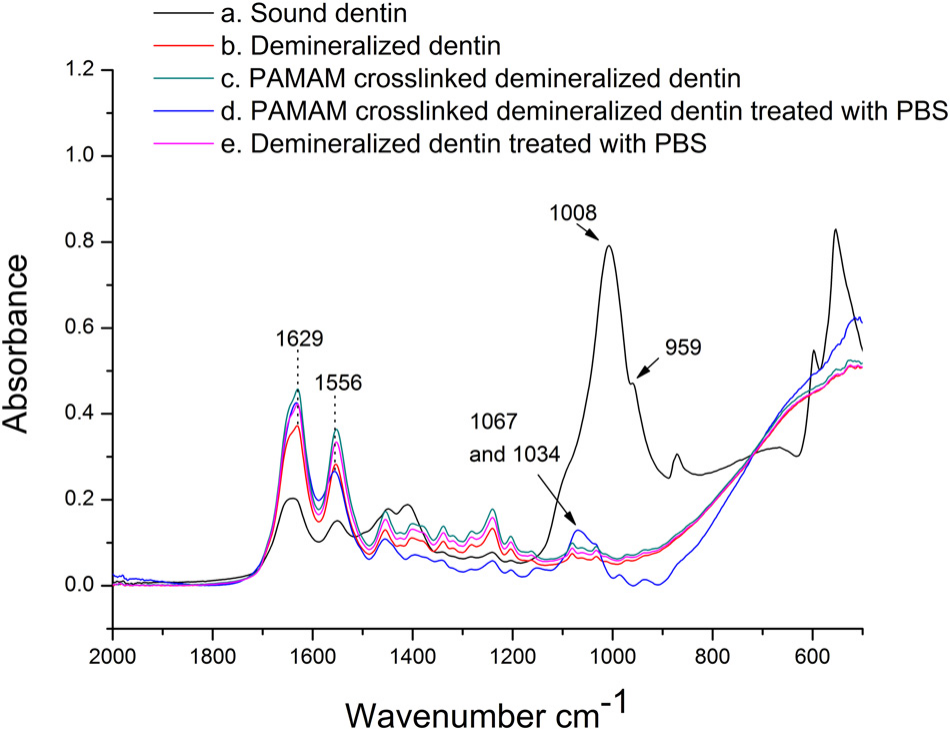
Infrared spectra (2,000–500 cm^-1^) of EDTA demineralized dentin discs crosslinked with PAMAM dendrimers. (a) Sound dentin (black line). (b) Dentin disc demineralized with 0.5 M neutral EDTA solution at room temperature for 72h (red line). (c) Demineralized dentin disc crosslinked with G3.0 PAMAM dendrimers using 0.25% glutaraldehyde at 4°C for 24h (green line). (d) PAMAM dendrimers crosslinked demineralized dentin disc immersed in pH 5.8 PBS for 2h (blue line). (e) Demineralized dentin disc immersed in pH 5.8 PBS for 2h without PAMAM dendrimer crosslinking (pink line).

Fig 2 showed the ATR-FTIR spectra of demineralized dentin discs before and after treated with G3.0 PAMAM dendrimer using glutaraldehyde as crosslinking agent. Changes after the treatment of PAMAM crosslinked dentin discs with PBS were also shown. Prior to demineralization, the resonances around 1000 cm^-1^ (1008 and 959 cm^-1^) were attributed to PO_4_^3-^(a). After the dentin discs were treated with neutral EDTA solution for 72 h, the ATR-FTIR analysis showed that the resonances at 1629 and 1556 cm^-1^, assigned as amide I and amide II bands of type I collagen[25], were strengthened, while the resonances attributed to PO_4_^3-^ were markedly weakened (b), indicating that the dentin discs were completely demineralized. After demineralized dentin discs were crosslinked with PAMAM dendrimer, the resonances assigned as amide I and amide II bands became more intense(c), indicating that PAMAM dendrimer molecules were successfully crosslinked to demineralized type I collagen fibrils. Compared to untreated demineralized dentin discs which were immersed in PBS for 2h (e), the appearance of new peaks assigned as PO_4_^3-^ (1067 and 1034 cm^-1^) after PAMAM crosslinked demineralized dentin discs were treated with PBS (d) indicated that PAMAM dendrimer molecules crosslinked to collagen fibrils of demineralized dentin discs had the capacity to attract PO_4_^3-^ through electrostatic interaction.

### Scanning electron microscopy and energy-dispersive X-ray spectroscopy analysis

**Fig 3:**
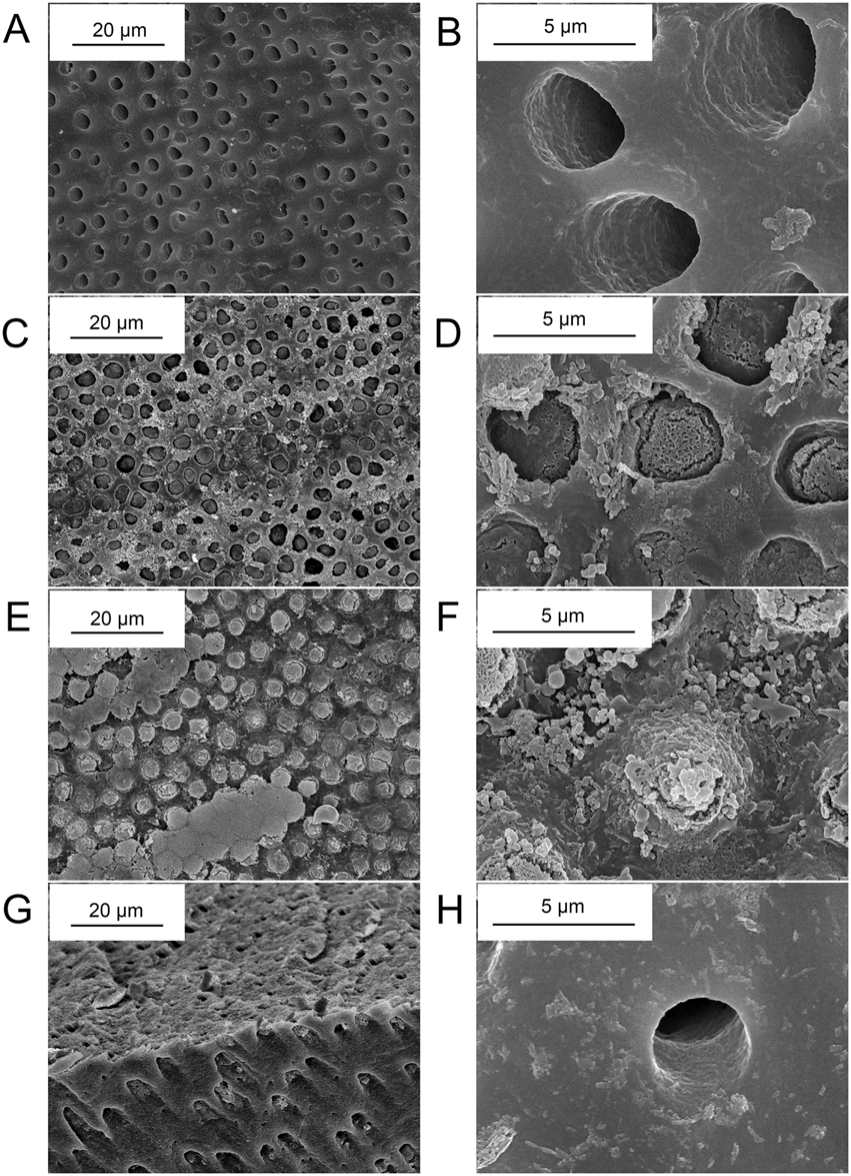
SEM images of dentin surface and longitudinal section morphology indicating dentinal tubules occlusion. (A, B) Dentin discs etched with 10% citric acid for 30s. All of the dentinal tubules were open. (C, D) Dentinal tubules occluded with newly formed rod-like crystals after dentin discs were coated with PAMAM dendrimers and immersed in SBF for 1w. The outermost part of tubules was left patent. (E, F) Dentinal tubules occluded with newly formed precipitates projecting form dentin surface. Part of the dentin surface was covered with globular and rod-like crystals. (G) Dentinal tubules occluded from the dentin surface to a depth up to approximately 20μm. (H) In control group, no significant difference was observed before and after dentin discs were directly immersed in SBF for 1w, without treated with PAMAM dendrimer.

The SEM image of dentin surface treated with 10% citric acid solution for 30 seconds showed that all dentinal tubules were open (Fig 3A and 3B), similar to hypersensitive dentin, indicating that the smear layer produced during cutting process was removed and the tubules were cleansed as a result of the citric acid treatment. The dentin tubules were completely free from debris. The peritubular dentin was compact and homogeneous. After dentin discs were coated with pure G3.0 PAMAM dendrimer and immersed in SBF at 37°C for 1 week, the open dentinal tubules were completely occluded with precipitates (Fig 3C–3F) and two kinds of typical appearances were observed. One was where the newly formed rod-like crystals sealed the dentinal tubules as plugs. However, the outermost part of tubules was left patent (Fig 3C and 3D). In the other case, dentinal tubules were occluded with newly formed precipitates projecting form dentin surface (Fig 3E and 3F). Globular and rod-like crystals were combined with each other and covered parts of the dentin surface. Fig 3G showed the longitudinal section parallel to the alignment direction of dentinal tubules after PAMAM coated dentin discs were immersed in SBF for 1 week. As shown in the figure, dentinal tubules were occluded from the dentin surface to a depth of approximately 5μm. In addition, precipitates were observed to have a better sealing effect in parts of dentinal tubules, reaching a depth of approximately 20μm. In the control group, no significant difference was observed before and after dentin discs were directly immersed in SBF for 1 week, without treated with PAMAM dendrimer (Fig 3H).

**Fig 4:**
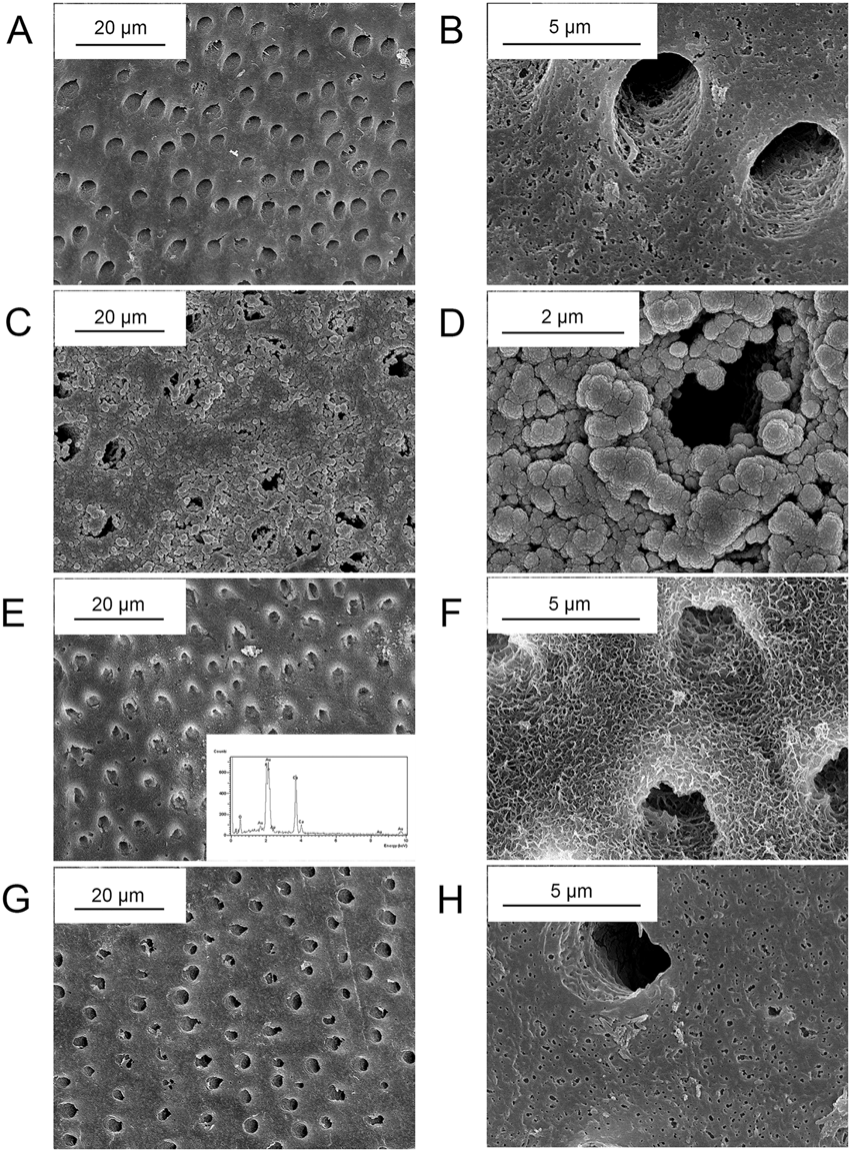
SEM images of the remineralization of demineralized dentin discs. (A, B) Dentin discs demineralized with neutral EDTA solution for 72h. (C, D) EDTA demineralized dentin discs crosslinked with G3.0 PAMAM dendrimers using 0.25% glutaraldehyde for 24h, exhibiting a “corn-on-the-cob” appearance. (E, F) Flake-like crystals were induced on the surface and covered the wall of dentinal tubules after PAMAM crosslinked dentin discs were treated with SBF for 1w, with dentinal tubules still left patent. (Insert of (E)) EDS showed the Ca/P molar ratio of the mineral crystals was 1.59±0.05. (G, H) No significant mineral crystals formed on the demineralized dentin discs after they were immersed in SBF for 1w, without treated with PAMAM dendrimers.

The surface morphologies of the demineralized dentin discs before and after crosslinked with PAMAM dendrimers were shown in Fig 4. As shown in Fig 4A and 4B, after the dentin discs were demineralized in neutral EDTA solution for 72 h, the smear layer was removed, the dentinal tubules were opened and the collagen fibrils were completely exposed. Collagen fibrils were thickened and embedded with spherical particles after demineralized dentin discs were treated with G3.0 PAMAM dendrimer using glutaraldehyde as crosslinking agent (Fig 4C and 4D). Flake-like crystals were induced on the surface of dentin discs and within dentinal tubules after PAMAM crosslinked dentin discs were treated with SBF for 1 week (Fig 4E and 4F). However, dentinal tubules were still patent although the walls of dentinal tubules were remineralized. X-ray spectroscopy (EDS) showed that the Ca/P molar ratio of the mineral crystals was 1.59±0.05 (Fig 4E insert), indicating that these mineral crystals were calcium-deficient HAP, as the Ca/P ratio of HAp is 1.67. Fig 4G and 4H showed that no significant mineral crystals formed on the demineralized dentin discs after they were immersed in SBF at 37°C for 1 week, without treated with PAMAM dendrimer.

### X-ray diffraction

**Fig 5:**
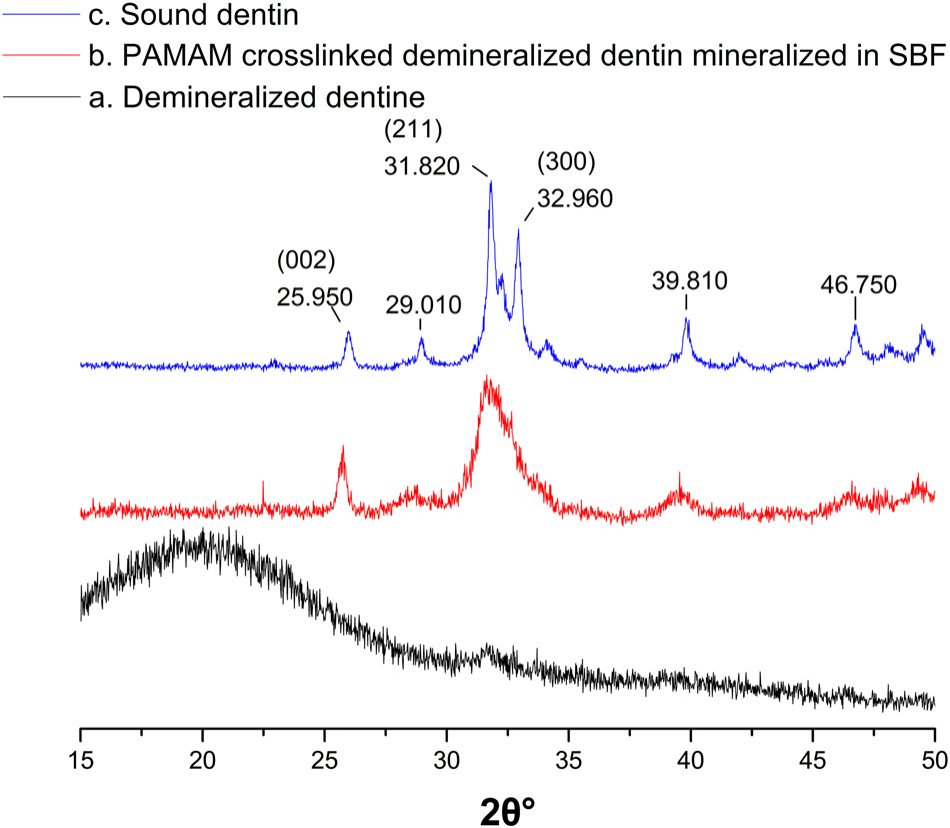
XRD spectra of EDTA demineralized dentin discs crosslinked with PAMAM dendrimers. (a) Neutral EDTA demineralized dentin (black line). (b) PAMAM dendrimers crosslinked demineralized dentin mineralized in SBF for 1w (red line). (c) Sound dentin (blue line).

Fig 5 showed the XRD results of the surface of the demineralized dentin discs, before and after they were crosslinked with PAMAM dendrimers. (XRD measurements of PAMAM coated dentin discs before and after they were immersed in SBF for 1w were not carried out because the surface of dentin discs which was not covered by mineral crystals was suggested to interfere with the results.) Fig 5a shows the XRD pattern of the demineralized dentin discs. The broader and shorter peaks between 30° and 35° indicate that the inorganic phase on the surface of the demineralized dentin discs lacked a crystal lattice. The broader peak at 20° indicates the presence of collagen. The XRD pattern of the biomimetically remineralized dentin discs (Fig 5c) shows the characteristic diffraction peaks of HAP, with a peak corresponding to the (0 0 2) plane and overlapping peaks corresponding to the (2 1 1), (1 1 2) and (3 0 0) planes[26–28]. This result is very similar to the XRD pattern of sound dentin that is shown in Fig 5b.

## Discussion

Occlusion of patent dentinal tubules is one of the main strategies in treating dentin hypersensitivity and has been extensively used to study the efficacy of desensitizing products[29–32]. Although a large number of in-office and over-the-counter (OTC) products have been shown to occlude dentinal tubules *in vitro* and *in vivo*[6–10], up to date, an ideal material that can completely treat dentin hypersensitivity and fulfill the requirements proposed by Grossman in 1935 has yet to be discovered[33]. Therefore, novel materials and methods are still needed to overcome the problems such as poor effectiveness and short durability encountered during the treatment of dentin hypersensitivity. Occlusion achieved by minerals which are induced by desensitizing agents within dentinal tubules is thus a feasible solution towards such problems. With the application of calcium phosphate precipitate method (CPP method) developed by Ishikawa et al. dentinal tubules could be successfully occluded with dicalcium phosphate dehydrate (DCPD; CaHPO_4_•2H_2_O) which might further transform to HAP with the addition of fluoride[13,15]. Ammonium hexafluorosilicate was also employed by the same research group and completely occlusion of patent dentinal tubules was achieved with the precipitation of silica-calcium phosphate which was well know to induce apatite formation from simulated body fluids[11,16]. Mesoporous biomaterials were also demonstrated to have superior performance in occluding dentinal tubules. Mesoporous silica nanoparticles (MSNs) embedded with Ca^2+^ or PO_4_^3-^ respectively could act as calcium and phosphate sources for the precipitation within dentinal tubules[17]. Nano CaO@mesoporous silica (NCMS) mixed with phosphoric acid was also demonstrated to reduce dentin permeability significantly, arising from the relatively quick precipitation and the tight sealing of the dentinal tubules[18]. Similarly, in the present study, a promising mineralization inducing agent, Poly(amidoamine) (PAMAM) dendrimer was employed and a more productive dentinal tubules occlusion through *in situ* mineralization was achieved by using the third generation (G3.0) molecules of it.

PAMAM dendrimers are regarded as artificial proteins on account of their well-defined structure and functional groups. The synthesis of PAMAM dendrimers is a highly branching process which initiates from an ethylenediamine (EDA) core and then forms a tree-like architecture distinguished by exponential numbers of discrete dendritic branches radiating out from the EDA core and leaves well-defined numbers of tertiary amino and primary amino groups in the interior and on the surface of the dendrimer molecule, respectively[19,34]. These amino groups could be easily transferred to other kinds of functional groups which would be valuable for mineralization. PAMAM dendrimers with modified surface groups have already been reported to induce mineralization under simulated physiological conditions. PAMAM with L-glutamic acid groups on the periphery was reported by Xie et al. to affect the crystallization of HAP[35]. Yang et al. successfully synthesized an amphiphilic PAMAM dendron with aspartic acids on the periphery and an aliphatic chain at the focal point and used it to regulate the process of HAP crystallization[36]. In addition, the capacity of amino group itself to induce mineralization in a pure supersaturated solution or a simulated body fluid (SBF) makes it possible for unmodified whole generation PAMAM dendrimers which contain large number of primary amino groups to be a promising option in occluding dentinal tubules with *in situ* formed HAP[37,38]. The results of this study demonstrated that PAMAM dendrimers trapped on the surface of dentin specimens or infiltrated into dentinal tubules had the ability to attract PO_4_^3-^ which was of great importance in the following mineralization process. Precipitate induced by PAMAM dendrimers within dentinal tubules could efficiently occlude dentinal tubules at both the exterior open end and in the depth (20μm) of dentinal tubules. The strong adherence between dentin and PAMAM dendrimers could be attributed to the flexibility of dendrimer molecules, the mucosity of the thickened liquid composed of amine terminated PAMAM dendrimers which can lead to the localization of PAMAM dendrimers to dentin surface and the positively charged nature of whole generation PAMAM dendrimers which makes it possible for them to interact with negatively charged sites of hydroxyaptite crystals. The liquid nature of PAMAM dendrimer would also help it to induce the nucleation and growth of mineral crystals available for effective dentinal tubules occlusion by infiltrating to a deeper depth in dentinal tubules, compared to other kinds of desensitizing agents, especially particles like nano-hydroxyapatite and MSN. The excellent biocompatibility and lower cytotoxicity of PAMAM dendrimers could lead to the future application of them in patients’ oral cavities[39,40].

Further, the ability of PAMAM dendrimer in inducing dentin remineralization was determined. Teeth are the most heavily mineralized tissues in human body. Demineralization and remineralization of teeth coexist during the entire life of an individual. Dental caries is a dynamic process caused by the imbalance between demineralization and remineralizaiton[41]. Although fluoride-releasing restorative materials are used to restore this imbalance and remineralize affected enamel, remineralization of dentin is more difficult to achieve and still needs the development of innovative method[42]. Resin-dentin bonding is another major reason for dentin demineralization. Although bonding of resin-based composite restorations has been revolutionized by continuing advances in dentin bonding technology over the past two decades, the short durability arising from the degradation of resin infiltrated collagen matrix caused by enzymes like matrix metalloproteinases (MMP) at the bonding interfaces is still a problem exigent to be solved[43]. As the collagen fibrils are exposed by etching with acids or acidic resin monomers derived from self-etching primers/adhesives, remineralization of demineralized collagen fibrils is thus worth of application in improving dentin bonding stability.

**Fig 6:**
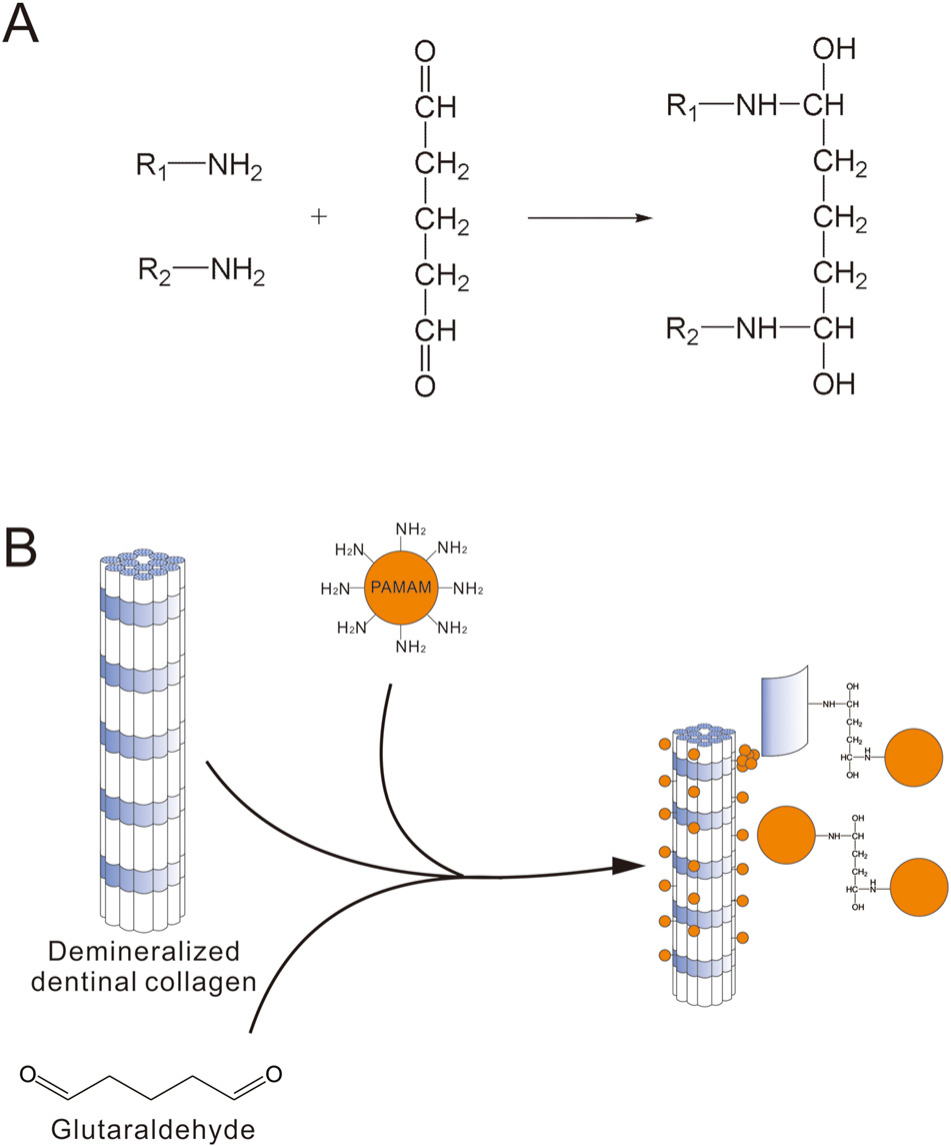
Mechanism of glutaraldehyde crosslinking. (A) Chemical reaction equation. (B) Schema chart that reveals the process of PAMAM dendrimers crosslinked to demineralized dentinal collagen by using glutaraldehyde.

To date, two main strategies were carried out to remineralize demineralized dentin. One strategy depended on the heterogeneous nucleation which was induced by seed crystallites arising from the partially demineralization. Although employed in many aspects including the remineralization of carious dentin, such a traditional ion-based strategy cannot be used in locations where seed crystallites are absent, for example the completely demineralized dentin created by etch-and-rinse adhesive systems or the superficial part of a caries-affected dentin lesion[44–46]. The other kind of remineralization strategy based on the bottom-up mechanism referring to material assembly from nanoscopic scale, such as molecules and atoms, to form larger structures[47]. Biomineralizaiton, especially the mineralization of human dentin, is a perfect example representing the bottom-up approach[48,49]. Dentin is a highly complex composite that is composed of 50 weight percent (wt%) inorganic mineral, 40 wt% extracellular matrix (ECM) and 10 wt% aqueous fluids[50]. Ninety weight percent of ECM is type I collagen which provides the three-dimensional structural framework for dentin biomineralization[51,52]. In addition to type I collagen, the ECM component contains acidic noncollagenous proteins (NCPs), including dentin matrix protein 1 (DMP-1), dentin sialaprotein (DSP) and dentin phosphoprotein (DPP)[53]. These NCPs are rich in acidic amino acids, such as glutamic acid and aspartic acid, which can attach to collagen fibrils, nucleate the formation of HAP, and regulate the dentin biomineralization process. Under regulation by NCPs, collagen fibrils are embedded with HAP platelets that are preferentially [0 0 1] aligned parallel to the long axis of the collagen fibrils, along the microfibrillar spaces; this forms the most fundamental level of the dentin structure[54]. In this study, PAMAM dendrimers possessing the ability to induce mineralization has been employed as a mimetic analog of NCPs. The occlusion of dentinal tubules was expected to be achieved through the further crystal growth after PAMAM induced nucleation. Different from the applications of other mimetic analogs like polyacrylic acid (PAA) and polyaspartic acid (pAsp) via electrostatic interaction, PAMAM dendrimers were crosslinked with demineralized dentin collagen using glutaraldehyde. Glutaraldehyde is the main ingredient in Gluma, which is a commonly used desensitizing agent. In this study, the crosslinking ability of glutaraldehyde was employed. Free primary amine groups (–NH_2_) of G3.0 PAMAM dendrimers and collagen fibrils can easily react with the aldehyde groups (-C = O) of glutaraldehyde to form Schiff’s base and the two kinds of molecules will be linked with covalent bonding[55] which can also be formed between PAMAM dendrimer molecules (Fig 6) and is better than easily interfered electrostatic interaction employed in the applications of other kinds of biomimetic analogs of NCPs (e.g. polyvinylphosphonic acid, PVPA)[56]. As shown in Fig 4C and 4D, demineralized collagen fibrils exhibited a “corn-on-the-cob” appearance after they were crosslinked with G3.0 PAMAM dendrimers using glutaraldehyde. With PAMAM dendrimers immobilized on the surface, a whole demineralized collagen fibril would turn into a nucleator and induce crystal formation in simulated body fluid. After crystal nucleation and PAMAM dendrimers crosslinked collagen fibrils were embedded with crystal nucleus, the surface of demineralized dentin and dentinal tubules would be covered with minerals which were induced through further heterogeneous nucleation, as is shown in SEM images. Although occlusion of dentinal tubules was not observed directly, remineralization of demineralized induced by PAMAM dendrimers immobilized on collagen fibrils is considered to have the potential to occlude patent dentinal tubules through the outward growth of the previously formed crystals. On the other hand, with glutaraldehyde, a network crosslinking structure can be created intra- and intermolecularly within collagen fibrils, providing the fibrillar resistance against enzymatic degradation as well as greater tensile properties, which is an important application in restorative dentistry[57].

Although there are still some drawbacks: (1) PAMAM dendrimers cannot be used directly in immediate occlusion of dentinal tubules as the sealing effect comes from the capacity of them in inducing mineralization within dentinal tubules which will be a relatively slow process costing up to 7 days, revealed in this study, otherwise the symptoms will last for a period of time instead of being relieved immediately when PAMAM dendrimers are applied in the treatment of dentin hypersensitivity, which will not be accepted by patients suffering from intolerable discomfort and (2) glutaraldehyde has a high cytotoxicity that may arise from residues of unreacted or degraded crosslinking agent, effective dentinal tubule occlusion was obtained successfully in this study. These drawbacks would be solved by including potassium ions and by replacing glutaraldehyde with other crosslinking agents of better biocompatibility and lower cytotoxicity in the future. Research related is under progress now.

## Conclusions

In conclusion, the null hypothesis must be partially rejected. Within the constraints of this experimental design, the following conclusions can be drawn:

1. After applied directly on the surfaces of dentin discs, G3.0 PAMAM dendrimers could infiltrate in dentinal tubules and bind to dentinal mineral crystals through adhesion and electrostatic interaction. Patent dentinal tubules could be totally occluded then after the nucleation and growth of crystals induced by G3.0 PAMAM dendrimers.

2. G3.0 PAMAM dendrimers could be employed as mimetic analogs of NCPs after they were crosslinked to demineralized collagen fibrils using glutaraldehyde. The formation of crystal nucleus followed by heterogeneous nucleation of crystals was considered to be applied in different aspects in dentistry, especially in the occlusion of dentinal tubules and the treatment of dentin hypersensitivity.
